# AKT3 deficiency in M2 macrophages impairs cutaneous wound healing by disrupting tissue remodeling

**DOI:** 10.18632/aging.103051

**Published:** 2020-04-14

**Authors:** Song Gu, Hanhao Dai, Xilian Zhao, Chang Gui, Jianchao Gui

**Affiliations:** 1 Department of Sports Medicine and Joint Surgery, Nanjing First Hospital, Nanjing Medical University, Nanjing, P.R. China; 2 Trauma Center, Shanghai General Hospital, Shanghai Jiaotong University School of Medicine, Shanghai, P.R. China; 3 Department of Biomedical Engineering, Washington University, St. Louis, MO 63130, USA

**Keywords:** cutaneous wound healing, M2 macrophage, tissue remodeling, AKT3, AKT signaling

## Abstract

AKT signaling and M2 macrophage-guided tissue repair are key factors in cutaneous wound healing. A delay in this process threatens human health worldwide. However, the role of AKT3 in delayed cutaneous wound healing is largely unknown. In this study, histological staining and transcriptomics demonstrated that prolonged tissue remodeling delayed wound healing. This delay was accompanied by defects in AKT3, collagen alpha-1(I) chain (COL1A1), and collagen alpha-1(XI) chain (COL11A1) expression and AKT signaling. The defect in AKT3 expression was M2 macrophage-specific, and decreased AKT3 protein levels were observed in CD68/CD206-positive macrophages from delayed wound tissue. Downregulation of AKT3 in M2 macrophages did not influence cell polarization but impaired collagen organization by inhibiting COL1A1 and COL11A1 expression in human skin fibroblasts (HSFs). Moreover, a co-culture model revealed that the downregulation of AKT3 in the human monocytic cell line (THP-1)-derived M2 macrophages impaired HSF proliferation and migration. Finally, cutaneous wound healing in AKT3^-/-^ mice was much slower than that of AKT3^+/+^ mice, and F4/80 macrophages from the AKT3^-/-^ mice had an impaired ability to promote wound healing. Thus, the downregulation of AKT3 in M2 macrophages prolonged tissue remodeling and delayed cutaneous wound healing.

## INTRODUCTION

Chronic wound healing occurs when a wound cannot restore the anatomical and functional integrity of the skin through a normal, orderly, and timely repair process, resulting in delayed wound healing [1]. The prevalence of various chronic diseases (diabetes) increases each year, leading to a higher incidence of associated chronic wounds. Although chronic refractory wounds are not immediately life-threatening, the delay in wound healing (e.g., months or years) has serious effects on a patient’s recovery from the primary disease and quality of life. It is also a significant burden on a patient’s family, both financially and as caregivers [2]. Cutaneous wound healing includes inflammation, tissue regeneration, and tissue remodeling that involves a complex orchestration of resident stem cells, immune cells, cytokines, and the extracellular matrix (ECM) [3, 4]. However, imbalances or defects in these processes can perturb the delicate equilibrium of cells and signaling pathways that are necessary for complete tissue repair. These defects can result in chronic wounds and fibrotic scars that impair normal tissue function, leading to organ failure and death [5]. Tissue remodeling was considered to be the final step of cutaneous wound healing and also closely related to well wound closure [6]. ECM components provide a “scaffold” for different cell types involved in tissue remodeling that are essential for the tissue repair process [7].

Complete wound healing and functional restoration of damaged skin in adults remain a significant challenge. Recent studies have demonstrated that the microenvironment, which consists of diverse immune cells, is crucial for human cutaneous wound healing, especially for tissue remodeling [8]. The immune system is an active component of tissue repair and regeneration. At the initiation and terminal stages of wound healing, the injured skin requires the activation of an immune response, which is characterized by the massive recruitment of immune cells [9]. Among the various immune cell types, macrophages, particularly those of the M2 phenotype (i.e., M2 macrophages), play a predominant role in tissue remodeling [10]. It is noteworthy that an alternative macrophage phenotype (M2 macrophage) is educated by the microenvironment at the injury site [11–14]. Recent studies showed that M2 macrophages encourage constructive tissue remodeling due to their capacity to remodel the ECM and synthesize multiple cytokines and growth factors [5]. A lack of M2 macrophages during tissue remodeling leads to delayed wound healing [5]. Although tissue repair is orchestrated by numerous cell types, macrophages are involved at all stages of the wound repair response and, thus, have emerged as potentially important therapeutic targets [10, 15, 16]. Although the importance of macrophages in cutaneous wound healing is clear, the specific molecular mechanism underlying the role of M2 macrophages in this process is unknown.

Signal transduction and activation are crucial for mammalian biological processes, such as growth, metabolism, angiogenesis, and wound healing [17]. AKT signaling plays a critical role in wound healing. Disrupted AKT signaling prolongs corneal epithelial wound healing by inhibiting epithelial cell proliferation [18]. The AKT pathway is also important for regulating macrophage survival, migration, and proliferation and orchestrating the responses of macrophages to different metabolic and inflammatory signals [19, 20]. AKT, also known as protein kinase B, is a key component of the PI3K/AKT signaling pathway. There are three AKT isoforms (i.e., AKT1, AKT2, and AKT3), which are responsible for different biological processes [21]. There is growing evidence that these isoforms are not redundant and have partially opposing effects [22]. Among the AKT isoforms, AKT3 is responsible for homeostasis and is frequently targeted by microRNA post-transcriptionally to attenuate peripheral nerve injury [23, 24]. Genetic ablation of AKT3 in macrophages promotes foam cell formation and atherosclerosis in mice [25]. We hypothesized that AKT3 might affect cutaneous wound healing through AKT signaling and AKT3-mediated M2 macrophage reprogramming.

In the current study, we found that both prolonged tissue remodeling and downregulated AKT3 expression occurred during delayed cutaneous wound healing. M2 macrophages derived from delayed wound tissue lacked AKT3 and were incapable of promoting human skin fibroblast (HSF) proliferation and migration. Genetic ablation of AKT3 in mice delayed cutaneous wound healing, specifically at the tissue remodeling stage.

## RESULTS

### Extracellular matrix remodeling and re-epithelialization was weaker in delayed wound tissue

**Figure 1:**
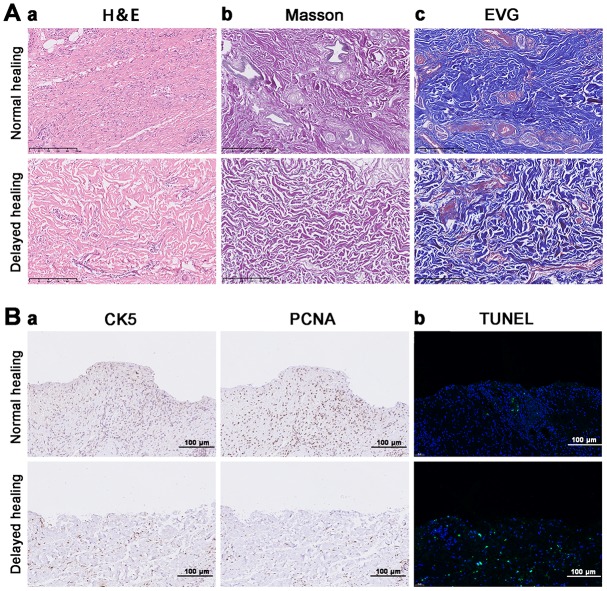
**Impaired tissue remodeling and re-epithelization in delayed cutaneous wound tissue.** (**A**) Histological staining of cutaneous wound tissue (200×). (**a**) H&E staining of sustained inflammatory cells and disordered tissue organization in the delayed cutaneous wound. (**b**) Masson staining of collagenous (blue) and muscular (red) fibers. Less staining was observed in the delayed wound tissue. (**c**) EVG staining of elastic fibers. Less staining occurred in the delayed wound tissue. (**B**) IHC and TUNEL staining of cutaneous wound tissue (200×). (**a**) CK-5 and PCNA expression levels are reduced in the delayed wound tissue. (**b**) Increased apoptosis (green cells) occurred in the delayed wound tissue. All the experiments were repeated at least three times.

Extracellular matrix components (e.g., fibronectin, elastin, and collagen) are essential for wound repair [26]. The restoration of tissue integrity is the result of neutrophils, monocytes, macrophages, fibroblasts, endothelial cells, and keratinocytes and the scaffold provided by the ECM [7, 27]. We found that tissue from cutaneous wounds with delayed healing had more inflammatory cells compared to tissue from wounds with a normal rate of healing (Figure 1A-a). Masson and EVG staining demonstrated that delayed wound tissue had significantly less assembled collagenous, muscular, and elastic fibers (Figure 1A-b). Re-epithelialization is another essential process in wound healing [28]. IHC staining with the re-epithelialization marker CK5 revealed that re-epithelialization was not complete in the delayed wound tissue, and PCNA expression on the delayed wound tissue surface was decreased compared to normal wound tissue (Figure 1B-a, b). There was also an increased number of apoptotic cells in the delayed wound tissue compared to the normal wound tissue (Figure 1B-c).

**Figure 2:**
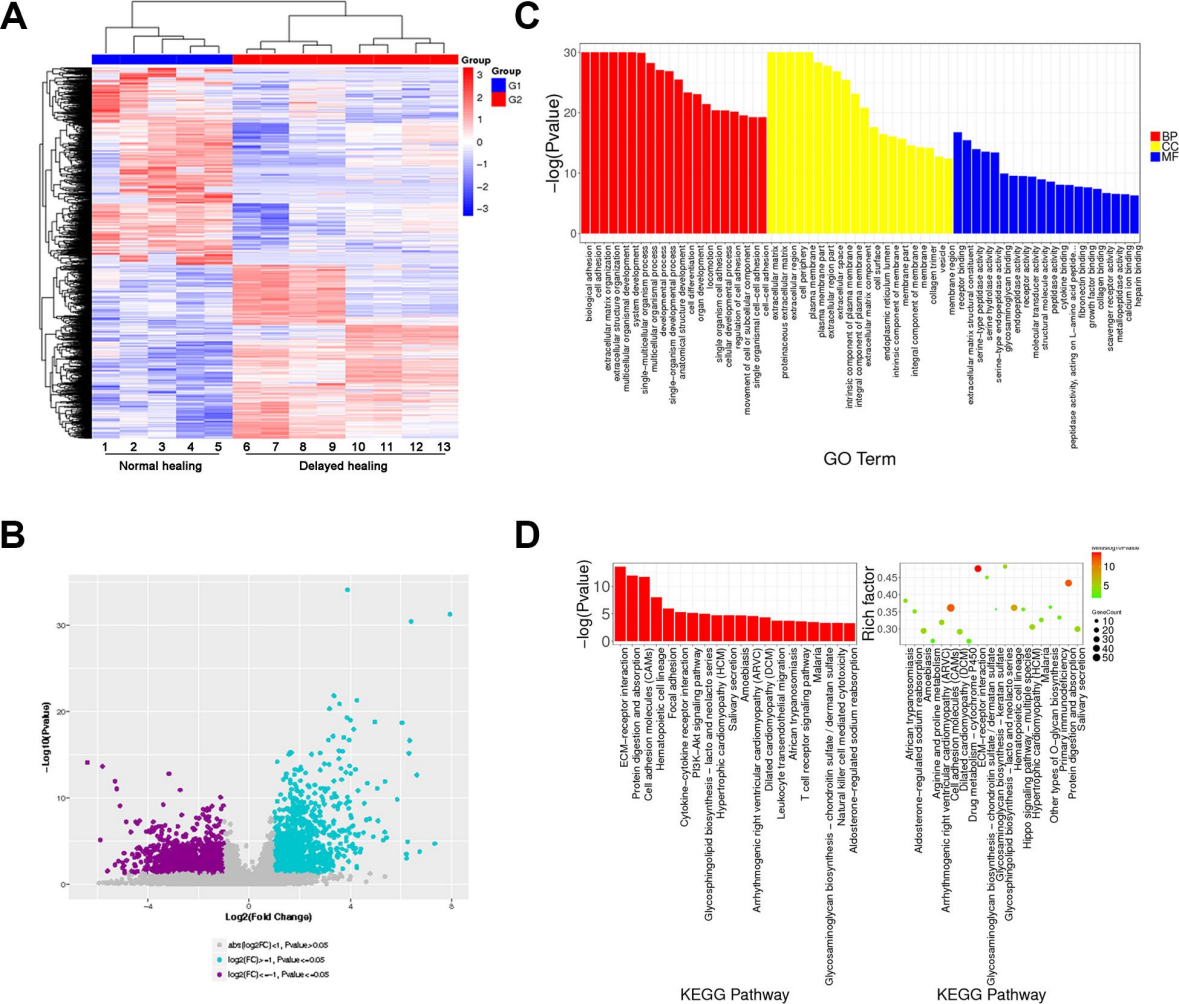
**Transcriptome analysis for normal and delayed cutaneous wound tissue.** (**A**, **B**) Cluster analysis for cutaneous wound tissue (normal wound tissue, n = 5; delayed wound tissue, n = 8). (**A**) Heatmap and (**B**) volcano plot for cutaneous wound tissue collected 28 days post-injury. (**C**) Gene expression profiles of normal and delayed cutaneous wound tissue generated by gene ontology (GO) analysis. (**D**) KEGG pathway analysis showed that the ECM-associated pathway was significantly enriched. Abbreviation: BB, biological process; CC, cellular component; MF, molecular function. All the experiments were repeated at least three times.

To explore the mechanism of delayed wound healing and re-epithelialization, RNA sequencing with clustering analysis (heatmap and volcano) identified a total of 1792 downregulated genes and 1570 upregulated genes (Figure 2A, 2B). Gene ontology (GO) and KEGG pathway analysis showed that the top 20 enrichment functions included ECM organization, interaction, and cell adhesion (Figure 2C, 2D). These results demonstrated that the assembly of ECM-associated collagenous, muscular, and elastic fibers was weakened, and the re-epithelization process was slowed in tissues with delayed wound healing.

### AKT3, COL1A1, and COL11A1 levels were downregulated in delayed wound tissue

**Figure 3:**
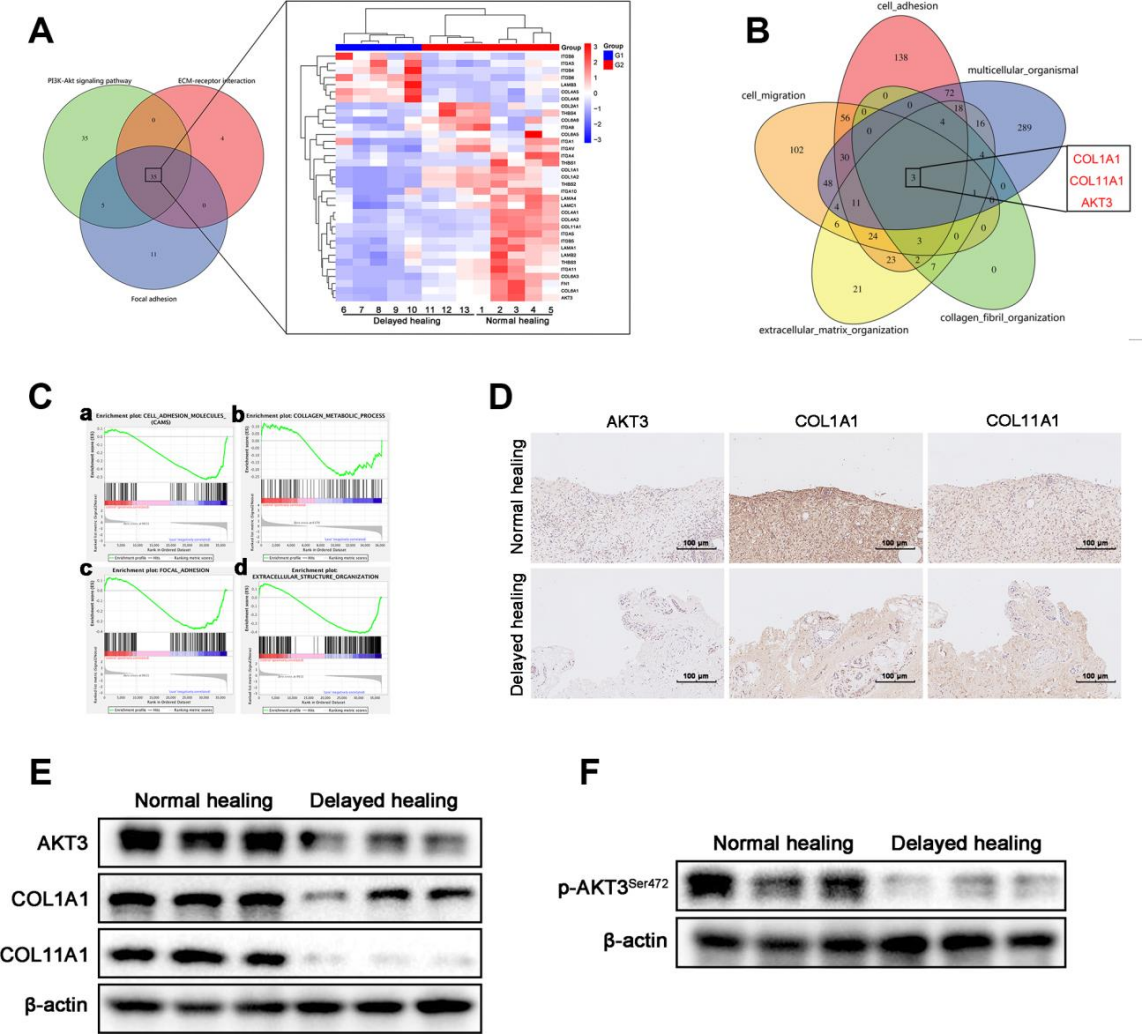
**Downregulation of AKT3, COL1A1, and COL11A1 in delayed cutaneous wound tissue.** (**A**) Venn diagram of the KEGG pathway. (**a**) Venn analysis identified 35 genes that were enriched in PI3K-AKT signaling, ECM-receptor interactions, and focal adhesion. (**b**) The heatmap expression profile of the 35 changed genes. (**B**) Venn diagram of GO analysis for the tissue remodeling-associated biological functions. AKT3, COL1A1, and COL11A1 were enriched. (**C** **a**–**d**) Gene set enrichment analysis (GSEA) of cutaneous wound tissue. The genes associated with (**a**) cell adhesion molecules, (**b**) collagen metabolic processes, (**c**) focal adhesion, and (**d**) extracellular structural organization were negatively enriched in the delayed cutaneous wound tissue. (**D**) IHC staining of AKT3, COL1A1, and COL11A1 in cutaneous wound tissue (200 x). The levels of all three proteins were reduced in the delayed wound tissue. (**E**) Decreased AKT3, COL1A1, and COL11A1 protein levels in delayed cutaneous wound tissue. (**F**) Total AKT3 and phosphorylated-Ser472 AKT3 levels were decreased in delayed cutaneous wound tissue. All the experiments were repeated at least three times.

To explore the molecular mechanism of impaired tissue remodeling, we evaluated three KEGG pathways associated with tissue remodeling, namely PI3K-AKT signaling, ECM-receptor interaction, and focal adhesion [7, 20]. These three KEGG pathways accounted for a total of 35 changed genes (Figure 3A). Based on the Venn data for the GO analysis, AKT3, COL1A1 (collagen type I alpha 1 chain), and COL11A1 (collagen type XI alpha 1 chain) were significantly enriched (Figure 3B). These data were consistent with the KEGG pathway analysis. Gene Set Enrichment Analysis (GSEA) was also used to analyze the tissue remodeling-associated gene set (Figure 3C a-d).

COL1A1 and COL11A1 are components of the ECM and play a pivotal role in the tissue remodeling phase of cutaneous wound healing [29, 30]. We evaluated AKT3, COL1A1, and COL11A1 expression in both normal and delayed wound tissue and found that expression of all three genes was decreased in the delayed wound tissue (Figure 3D, 3E, Supplementary Figure 2A). Activation of AKT signaling was also diminished in the delayed wound tissue, as indicated by weak phosphorylation at S472 of AKT3 (Figure 3F, Supplementary Figure 2B). These results suggested that AKT3, COL1A1, and COL11A1 were downregulated, and AKT3-related AKT signaling was deficient in delayed wound tissue.

### AKT3 deficiency in M2 macrophages caused downregulation of COL1A1 and COL11A1

**Figure 4:**
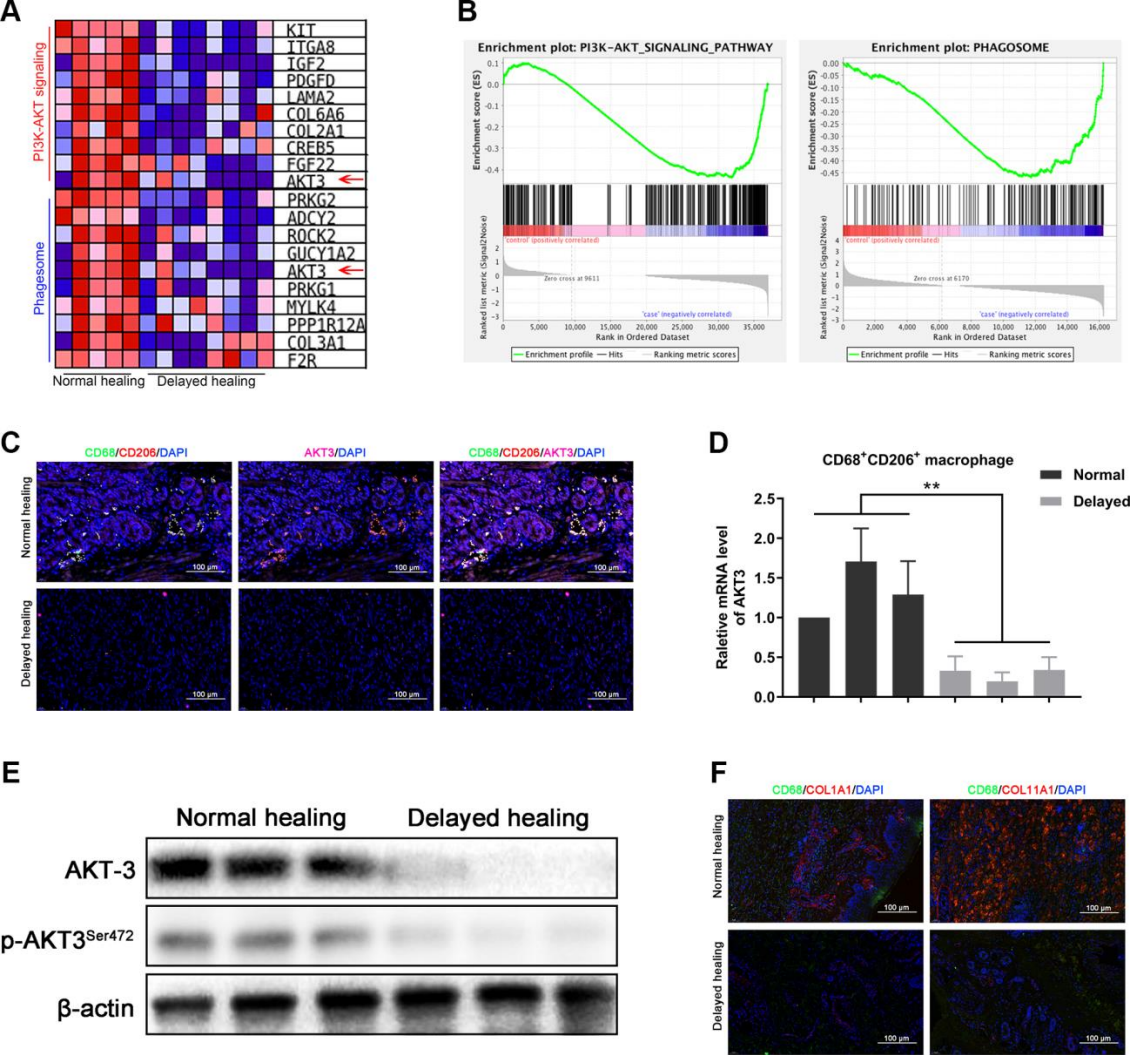
**Loss of AKT3 in M2 macrophages inhibited extracellular COL1A1 and COL11A1 expression.** (**A**) GSEA showed that negatively enriched genes were associated with PI3K-AKT signaling and phagosomes in delayed cutaneous wound tissue. (**B**) Heatmap of the top 10 genes related to PI3K-AKT signaling and phagosomes; AKT3 was downregulated in both functional enrichment sets in the delayed cutaneous wound tissue. (**C**) Immunofluorescence of cutaneous wound tissue (n = 6). CD68- (green) and CD206-(red) positive M2 macrophages were reduced in the delayed cutaneous wound tissue. AKT3 (pink) was decreased in the M2 macrophages. (**D**) qRT-PCR showed decreased AKT3 mRNA expression in the delayed cutaneous wound tissue-derived M2 macrophages. (**E**) Western blotting verified the reduction and loss of AKT3 in M2 macrophages from delayed cutaneous wound tissue. (**F**) Immunofluorescence of COL1A1 and COL11A1 in CD68-positive macrophages in cutaneous wound tissue. (**a**) Decreased CD68-positive macrophage infiltration and COL1A1 protein expression were observed in delayed cutaneous wound tissue. (**b**) Decreased COL11A1 protein expression also accompanied the reduced CD68-positive macrophage infiltration. All the experiments were repeated at least three times.

M2 macrophages are crucial for cutaneous wound healing and healing of wounds in other organs [10, 28]. AKT3 deficiency in M2 macrophages is responsible for cholesterol metabolism which was closely corelated to wound repair [25, 31]. We evaluated whether the downregulation of AKT3 was M2 macrophage-specific and responsible for the changes observed in COL1A1 and COL11A1 expression in the delayed wound tissue. GSEA revealed that PI3K-AKT signaling and phagosome-related genes (characteristic of macrophages) were negatively enriched in the delayed wound tissue (Figure 4A). The PI3K-AKT signaling and phagosome-associated heatmaps revealed that AKT3, which was one of the top 10 changed genes, was decreased in both the PI3K-AKT3 signaling and phagosome categories for the delayed wound tissue (Figure 4B).

Immunofluorescence staining for CD68, CD206, and AKT3 demonstrated that AKT3 was downregulated in CD68/CD206-positive M2 macrophages, and there was less infiltration of this macrophage population into the delayed wound tissue (Figure 4C). qRT-PCR confirmed that the sorted CD68/CD206-positive cells were M2 macrophages with decreased AKT3 mRNA levels (Figure 4D). In addition, total AKT3 and phosphorylated AKT3^S472^ protein levels were lower in the delayed wound tissue compared to the normal wound tissue (Figure 4E, Supplementary Figure 2C). Furthermore, decreased COL1A1 and COL11A1 protein levels were observed in the CD68-positive macrophages (Figure 4F a-b). These results suggested that AKT3 expression was specifically altered in M2 macrophages, leading to reduced infiltration into the wound and decreased COL1A1 and COL11A1 expression.

### AKT3 knockdown in M2 macrophages impaired HSF proliferation and migration in *ex vivo* co-culture model

**Figure 5:**
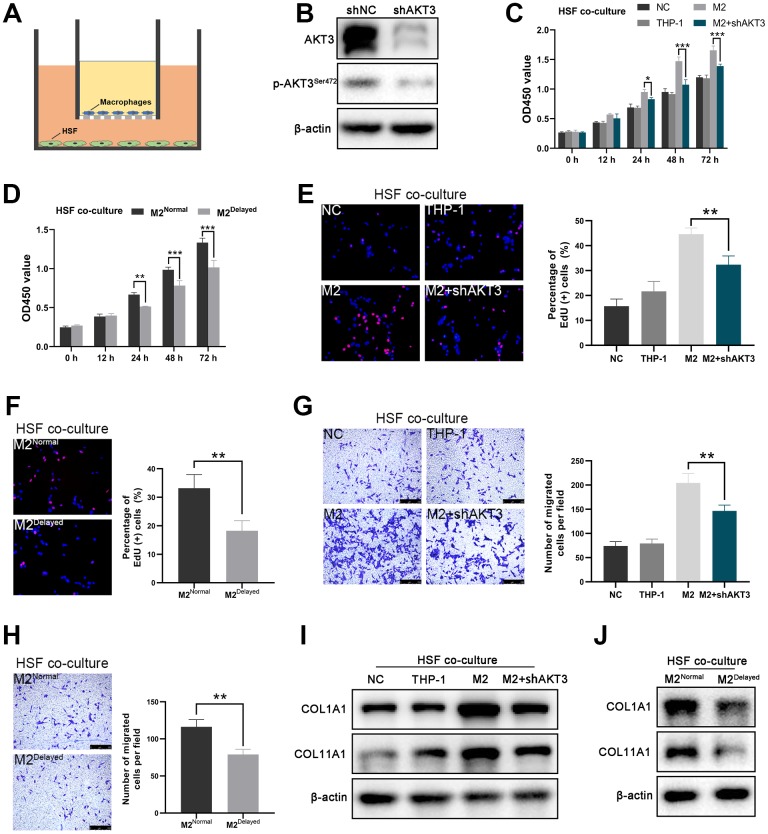
**AKT3 knockdown in M2 macrophages suppressed proliferation and migration as well as COL1A1 and COL11A1 expression *ex vivo.*** (**A**) Schematic of the M2 macrophage-HSF co-culture model. (**B**) Total AKT3 and associated phosphorylated AKT3^Ser472^ levels in THP-1-derived M2 macrophages following AKT3 knockdown. (**C**–**D**) CCK-8 assay of the co-culture model. (**C**) Proliferation of co-cultured HSFs was impaired following AKT3 knockdown in THP-1-derived M2 macrophages. (**D**) M2 macrophages isolated from delayed cutaneous wound tissue also lost their ability to facilitate HSF proliferation compared to M2 macrophages derived from normal wound tissue. (**E**, **F**) EdU assay of the co-culture model. (**E**) DNA replication induced by M2 macrophages in HSFs was abrogated by AKT3 knockdown in these macrophages. (**F**) M2 macrophages from delayed cutaneous wound tissue were incapable of promoting HSF DNA replication. (**G**, **H**) Transwell migration assay of the co-culture model. (**G**) HSF migration was impaired after co-culture with AKT3 knockdown in THP-1-derived M2 macrophages. (**H**) M2 macrophages isolated from delayed cutaneous wound tissue could not promote HSF migration. (**I**) COL1A1 and COL11A1 protein levels were increased in HSFs co-cultured with THP-1-derived M2 macrophages. AKT3 knockdown in the M2 macrophages decreased COL1A1 and COL11A1 expression in the co-cultured HSFs. (**J**) M2 macrophages from delayed cutaneous wound tissue were incapable of inducing COL1A1 and COL11A1 expression in co-cultured HSFs compared to normal wound tissue-derived M2 macrophages. All the experiments were repeated at least three times.

To further investigate the influence of AKT3 deficiency on M2 macrophages, we established a co-culture model of THP-1-derived M2 macrophages and HSFs using a transwell non-contact co-culture system (Figure 5A). QRT-PCR confirmed the differentiation of THP-1 cells into M2 macrophages (Supplementary Figure 1A). AKT3 knockdown with shAKT3 in M2 macrophages was verified by western blotting. Phosphorylation of AKT3^S472^ was also reduced in these M2 macrophages (Figure 5B).

In the *ex vivo* co-cultures, the M2 macrophages derived from the THP-1 cells significantly increased HSF proliferation, which was partially abolished by AKT3 knockdown (Figure 5C, 5E). In contrast, M2^Delayed^ macrophages failed to promote HSF proliferation compared to M2^Normal^ macrophages (Figure 5D, 5F). Cell migration and scratch wound healing are considered to be the effector of wound healing [34]. In this study, we tested the effect of M2 macrophage co-culture on the migration of HSFs in vitro. THP-1-derived M2 macrophages significantly increased HSF migration, which was partially abrogated by AKT3 knockdown in the M2 macrophages (Figure 5G). Not surprisingly, M2^Delayed^ macrophages did not promote HSF migration compared to M2^Normal^ macrophages (Figure 5H). Thus, the elimination of AKT3 expression in M2 macrophages impaired the proliferation and migration of co-cultured HSFs.

The presence of the THP-1-derived M2 macrophages dramatically increased COL1A1 and COL11A1 expression in the HSF cells; however, this effect was abolished by AKT3 knockdown in the M2 macrophages (Figure 5I, Supplementary Figure 2D). The patient-derived M2 macrophages had a similar effect on the HSFs. Lower COL1A1 and COL11A1 expression levels were observed in the HSFs exposed to M2^Delayed^ macrophages, which had decreased AKT3 levels compared to the M2^Normal^ macrophages (Figure 5J).

### AKT3 knockout impeded cutaneous wound healing *in vivo*

**Figure 6:**
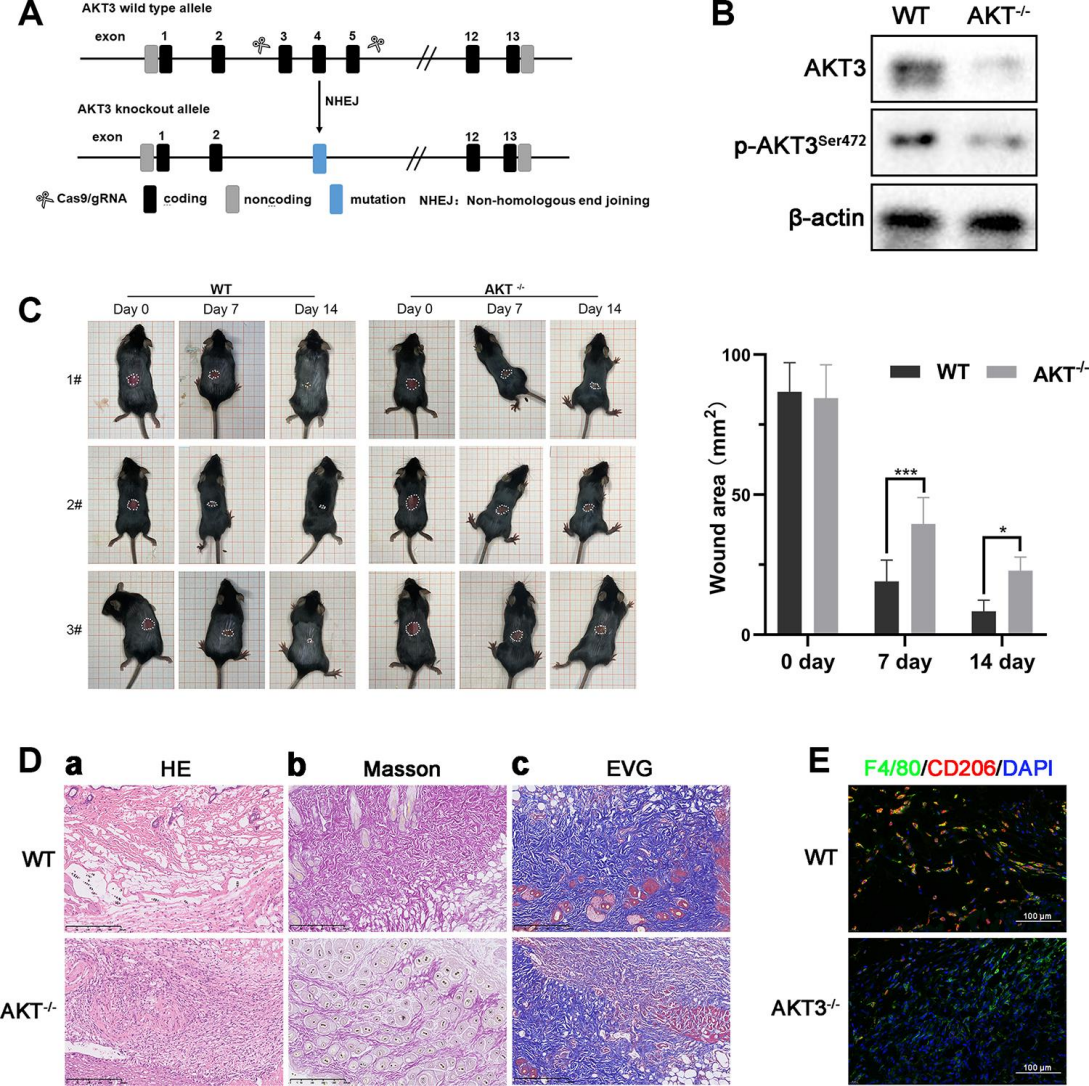
**Loss of AKT3 delayed cutaneous wound healing in mice.** (**A**) Schematic of AKT3 knockout in mice. (**B**) Western blotting for AKT3 levels in AKT3^+/+^ and AKT3^-/-^ mice (n = 6). (**C**) AKT3 knockout delayed cutaneous wound healing in mice by days 7 and 14 post-injury. (**D** **a**–**c**) Histological staining of mouse cutaneous wound tissue. (**a**) H&E staining showed more inflammatory cells in the wound tissue of AKT3^-/-^ mice and incomplete tissue integrity (n = 6). (**b**) Masson staining showed the numbers of collagenous and muscular fibers were reduced in the wound tissue of AKT3^-/-^ mice (n = 6). (**c**) EVG staining showed that number of elastin fibers were decreased in the wound tissue of AKT3^-/-^ mice (n = 6). (**E**) IF staining showed the F40/80 and CD206 expression in mouse cutaneous wound tissue. All the experiments were repeated at least three times.

To study the functional role of Akt3 in cutaneous wound healing in vivo, we used CRISPR/Cas9 technology to genetically ablate the AKT3 gene in C57BL/6 mice (Figure 6A). AKT3 expression and AKT3^S472^ phosphorylation were significantly reduced in the AKT3^-/-^ mice compared to the AKT3^+/+^ mice (Figure 6B). Interestingly, loss of AKT3 did not alter mouse body weight (Supplementary Figure 1B).

Therefore, we hypothesized that AKT3 might be involved in the regulation of cutaneous wound healing through its effects on M2 macrophage function. Although M2 macrophages are active in all stages of cutaneous wound healing, the most infiltration by these macrophages into the wound occurs during the tissue remodeling phase. We observed that on day 0 post-injury, the wound lesion area was similar between the AKT3^-/-^ and AKT3^+/+^ mice. By day 7 post-injury, the lesion areas of the AKT3^-/-^ mice were significantly larger than those of the AKT3^+/+^ mice. Not surprisingly, the lesions of the AKT3^+/+^ mice were almost healed by day 14 post-injury. In contrast, the lesions of the AKT3^-/-^ mice were almost the same size as they were on day 7 (Figure 6C). Histological analysis of the wound tissue showed that the wound structure in the AKT3^+/+^ mice demonstrated greater integrity and tightness compared to that of the AKT3^-/-^ mice. In addition, collagenous, muscular, and elastic fibers were more abundant in the wound lesion area of the AKT3^+/+^ mice compared to that observed for the AKT3^-/-^ mice (Figure 6D). AKT3 knockout also affected ECM deposition by inhibiting COL1A1 and COL11A1 expression (Supplementary Figure 1C, 1D(a)). Moreover, AKT3 knockout decreased M2 macrophage infiltration into the cutaneous wound site (Figure 6E, Supplementary Figure 1D(b)). These results suggested that loss of AKT3 delayed cutaneous wound healing by disrupting the tissue remodeling process.

**Figure 7:**
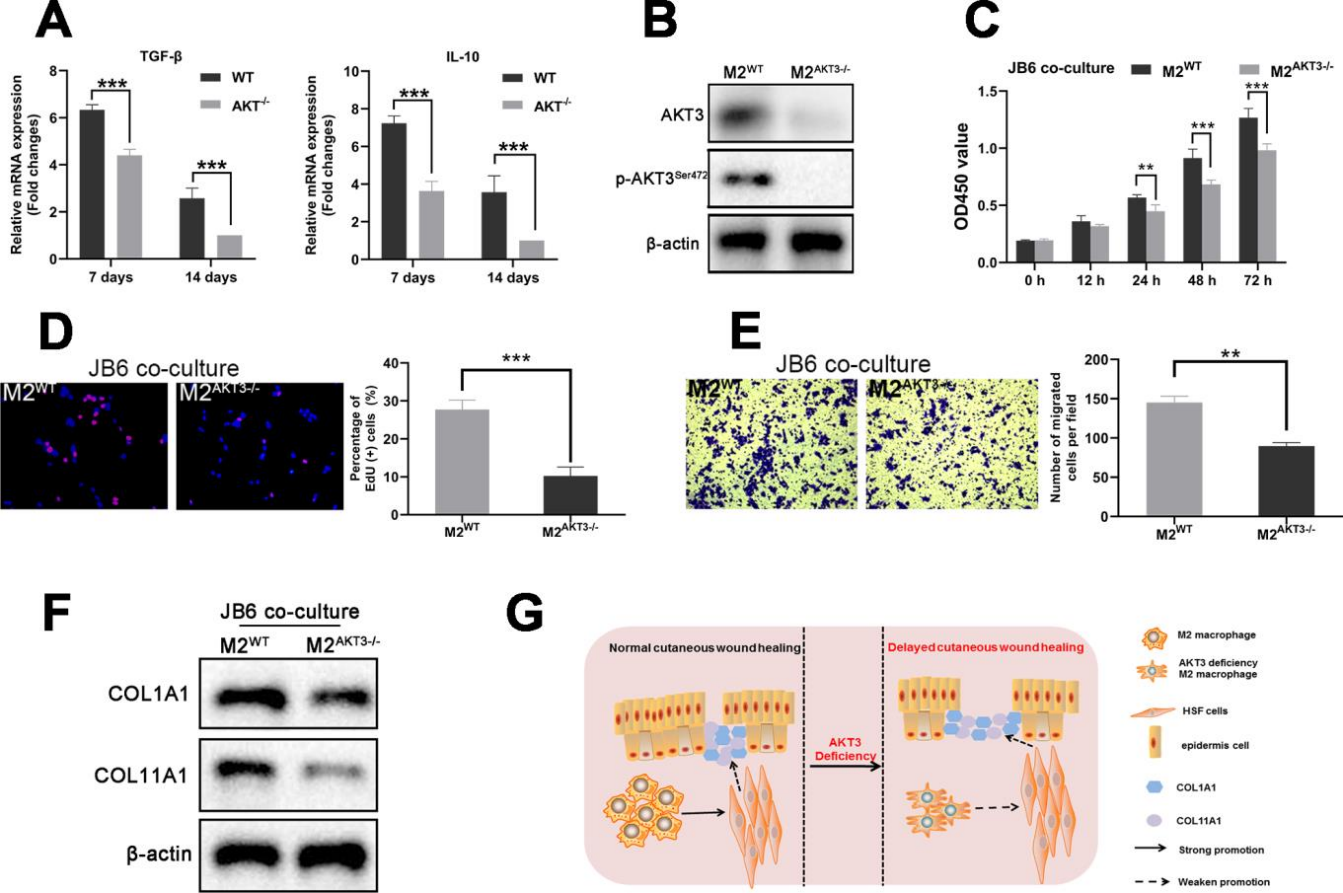
**M2 macrophages from AKT3^-/-^ mice failed to promote cell proliferation and migration *ex vivo.*** (**A**) TGF-β and IL-10 mRNA levels were decreased in delayed cutaneous wound tissue 7^th^ and 14^th^ day post-injury in mice (n = 3). (**B**) Western blotting demonstrated the loss of AKT3 in M2 macrophages from AKT3^-/-^ mice. (**C**, **D**) CCK-8 and EdU assays demonstrated that M2 macrophages from AKT3^-/-^ mice were incapable of promoting JB6 cell proliferation (**C**) or DNA replication (**D**), respectively. (**E**) Transwell migration assay showed M2 macrophages from AKT3^-/-^ mice could not promote JB6 cell migration. (**F**) COL1A1 and COL11A1 protein levels in JB6 were not increased by co-culture with M2 macrophages from AKT3^-/-^ mice. (**G**) The schematic illustration of the role of M2 macrophage AKT3 deficiency in delayed cutaneous wound healing. All the experiments were repeated at least three times.

During the tissue remodeling phase of wound healing, we observed that both TGF-β and IL-10 were expressed on days 7 and 14 post-injury, and the levels of expression were lower in the AKT3^-/-^ mice (Figure 7A). AKT3 expression levels in these F4/80/CD206-positive M2 macrophages were also verified by western blotting (Figure 7B). Previous research demonstrated keratinocyte meditated epidermal proliferation was crucial for cutaneous wound healing and was positively regulated by Erk/Akt signaling pathway [33]. To further investigate M2 macrophage to proliferation and migration of mouse JB6 cells, we co-cultured these mouse-derived F4/80/CD206 positive M2 macrophages with the JB6 murine epidermal cell line, results demonstrated that M2 macrophages derived from the wound tissue of AKT3^-/-^ mice lost their ability to promote cell proliferation and migration (Figure 7C–7E). COL1A1 and COL11A1 expression levels were also decreased in the JB6 cells co-cultured with M2 macrophages from the AKT3^-/-^ mice (Figure 7F). Overall, our study demonstrated that decreased M2 macrophage infiltration and impaired function were the underlying causes of delayed cutaneous wound healing. AKT3 deficiency in M2 macrophages appeared to be responsible for this abnormal M2 macrophage infiltration and function.

## DISCUSSION

Restoration of cutaneous integrity after an injury is of vital importance. Intact skin provides the first barrier against invading microbes and pathogens. Loss of the integrity of large portions of the skin as a result of injury or illness can lead to major disabilities and even death [34]. Cutaneous wound healing consists of three main phases (i.e., inflammation, regeneration, and remodeling), which are tightly linked [35]. Delayed cutaneous wound healing is frequently encountered with the large wound lesions of patients with diabetes, vascular disease, or dermatosis [36, 37]. The present study consisted of male patients with injury to a large portion of their legs. We observed the absence of skin integrity in the delayed wound tissue that was accompanied by reduced numbers of collagenous, muscular, and elastic fibers. It is well known that re-epithelialization of a wound plays a crucial role in maintaining cutaneous integrity [38, 39]. We observed slowed re-epithelialization in the delayed wound tissue. We also noticed that this wound tissue was incapable of proliferating compared to normal wound tissue. TUNEL staining suggested that there was excessive inflammation in the delayed wound tissue, which is consistent with data demonstrating that a chronic wound inflammatory response can contribute to delayed wound healing [40]. Our current results suggested that a disruption of the tissue remodeling phase may be the main cause of the delayed cutaneous wound healing.

We used next-generation sequencing to further investigate the molecular mechanisms involved in the impaired tissue remodeling observed with delayed cutaneous wound healing. We found that the ECM organization, ECM-receptor interactions, and cell adhesion were enriched. In particular, transcriptome analysis demonstrated that the expression of AKT3, COL1A1, and COL11A1 in delayed wound tissue was downregulated in three tissue remodeling-related processes, including PI3K-AKT signaling, focal adhesion, and ECM-receptor interaction. These changes were confirmed by IHC and western blotting. Based on these results, we asked whether the changes observed in AKT3, COL1A1, and COL11A1 expression were truly linked or just coincidental.

M2 macrophages are indispensable for cutaneous wound healing, especially during the tissue remodeling phase [10]. A deficiency in AKT3-related PI3K-AKT signaling can suppress the polarization of these macrophages [41]. In the present study, GSEA revealed that PI3K-AKT signaling and phagosome-related genes were negatively enriched in the delayed wound tissue, with AKT3 being downregulated in both gene sets. We observed reduced CD68/CD206-positive M2 macrophage infiltration in the delayed wound tissue, which was accompanied by decreased AKT3 expression in the M2 macrophage population and concomitant decreases in COL1A1 and COL11A1 expression in this tissue. Studies have shown that HSFs are the main sources of collagen in the skin [42]. Our current findings suggest that an AKT3 deficiency leads to the inability of M2 macrophages to induce COL1A1 and COL11A1 expression in cutaneous wounds (i.e., in HSFs). Indeed, AKT3 knockdown abolished M2 macrophage-induced increases in HSF proliferation and migration and COL1A1 and COL11A1 expression in our co-culture system.

In the present study, we failed to connect AKT3 directly to the expression of COL1A1 and COL11A1 in HSFs. Understanding the mechanism underlying this phenomenon requires further investigation. AKT3 is a vital component of the PI3K-AKT signaling pathway, which is crucial for androgen receptor regulation [43, 44]. However, the androgen receptor is negatively correlated with cutaneous and prostatic wound healing [45, 46]. Currently, we cannot exclude the influence of androgen on cutaneous wound healing in our study because the tissue samples that we analyzed were mainly obtained from middle-aged men. In addition, we need to analyze more wound tissue samples along with clinical data to clarify the relationship between low AKT3 levels and delayed cutaneous wound healing.

AKTs are a family of protein kinase B involved in cellular biology. Although they share some properties, it is clear that they each have distinct functions. AKT1 can enhance apoptosis and cell growth in mice [47], whereas AKT2 deficient mice have a diabetic phenotype and are resistant to insulin therapy [48]. In contrast, loss of AKT3 in mice induces foam cell formation and atherosclerosis [25]. Currently, very little is known about the role of AKT3 in delayed cutaneous wound healing. In the present study, we uncovered the potential of AKT3 deficiency to impair M2 macrophage infiltration and function in delayed cutaneous wound healing. Through the generation of an AKT3 knockout mouse model, we have identified a potential target for accelerating cutaneous wound healing in the absence of significant morbidity or death. Whether AKT3 plays a critical role in other M2 macrophage-associated biological processes is currently unknown. Future studies will evaluate the potential contribution of other AKT isoforms (AKT1 and AKT2) to delayed cutaneous wound healing. The classical and extensively activated PI3K/AKT/mTOR signaling pathway should also be included in the future research to clarify its function in cutaneous wound healing. Our transcriptome data suggest that these isoforms are unlikely to be involved in this process. Extensive studies have determined that AKT3 plays a role in the activation of the DNA repair pathway [49], suggesting that the involvement of AKT3 in tissue injury repair is potentially wide-ranging and not limited to cutaneous wounds.

## MATERIALS AND METHODS

### Patients and cell culture

The present study was approved by the ethics committee of Shanghai General Hospital. Crural wound tissue samples (n=13) were obtained from patients of Shanghai General Hospital. All samples were collected with informed consent. The inclusion criteria were as follows: male; 40 to 60 years of age; no diabetes; no vascular disease of the lower extremities; no nerve injury. Patients whose wound area showed no apparent reduction after standard treatment for one week were assigned to the delayed wound group. Patients that did not demonstrate delayed wound healing were assigned to the normal wound group. HSFs and JB6 cells were obtained from the Chinese Academy of Sciences Committee on Type Culture Collection Cell Bank (Shanghai, China). The HSFs and JB6 cells were cultured in Dulbecco’s Modified Eagle’s Medium (DMEM; Gibco, Grand Island, NY) supplemented with 10% fetal bovine serum (FBS) and maintained in 5% CO_2_ at 37°C. The human monocytic cell line THP-1 was purchased from the American Type Culture Collection (ATCC, Manassas, VA, USA) and cultured in RPMI 1640 medium with 10% FBS at 37°C and 5% CO_2_. THP-1 cells were differentiated into M2 macrophages, as previously described [28]. Briefly, THP-1 cells were incubated with 100 nmol PMA for 30 h to induce differentiation into macrophages. After washing three times, the adherent macrophages were treated with IL-4 (20 ng/mL) and IL-13 (20 ng/mL) to induce the CD68+/CD206+ M2 phenotype. For macrophage sorting, wound tissue samples were digested at 37°C. The samples were incubated with fluorescein isothiocyanate (FITC)-CD68 and phycoerythrin (PE)-CD206 or FITC-F4/80 antibodies for 40 min. After filtering through a 70-μm nylon cell strainer, the samples were analyzed using a Sony SH800 flow cytometer (Sony Biotechnology, Japan). Ten thousand events were collected using a forward scatter threshold of 50,000 (5%). Debris was excluded according to the FSC/SSC (forward scatter/side scatter) dot plot.

The establishment of in vitro macrophage co-culture model was followed the method which has been previously described [50]. THP-1 induced M2 macrophages and wound tissue sorted macrophages were seeded into Transwell chambers (Corning, New York, USA). The inserts with macrophages were put into the HSF or JB-6 cells, forming in vitro co-culture models to be applied in the following studies.

### Reagents and antibodies

Phorbol-12-myristate-13-acetate (PMA, cat no. HY-18739) were procured from MCE (Monmouth Junction, NJ, USA). Interleukin 4 (IL-4, cat no. AF-200-04-5) and Interleukin 13 (IL-13, cat no. AF-200-13-500) were purchased from Pepro Tech (Rocky Hill, NJ, USA). Antibodies were obtained commercially as below and diluted as recommendation in each experiment:

1. AKT3 (cat no. PA5-29696), COL1A1 (cat no. PA5-89281), COL11A1 (cat no. PA5-68410) and CD206 (cat no. MA5-16871) antibodies from Invitrogen (Carlsbad, CA, USA).
2. Antibodies against CK5 (cytokeratin 5) (cat no. 71536), PCNA (Proliferating Cell Nuclear Antigen) (cat no. 13110), COL1A1 (cat no. 39952), COL11A1 (cat no. 96321), β-actin (cat no. 3700), CD68 (cat no. 76437), F4/80 (cat no. 30325) from Cell Signaling Technology (Danvers, MA, USA).
3. Antibody against phoso-AKT3^ser472^ (cat no. orb6790) from Biorbyt.
4. Antibodies used in Fluorescence-Activated Cell Sorting (FACS) against CD68 (cat no. ab134351), CD163 (cat no. ab95613) and F4/80 (cat no. ab60343) from Abcam (Cambridge, MA).

### Histological analysis

Hematoxylin-eosin (H&E), Masson’s trichrome, and elastic Van Gieson (EVG) staining were performed to detect wound healing and collagen deposition in the wound tissue. Immunohistochemistry (IHC) was performed on tissue sections using antibodies against CK5, PCNA, AKT3, COL1A1, and COL11A1. Immunofluorescence (IF) was carried out with antibodies against CD68, CD206, F4/80, COL1A1, and COL11A1 and observed using fluorescence microscopy. To be specific, formalin-fixed, paraffin-embedded tissue samples were cut into 4-μm-thick sections. 0.01 M citrate buffer (pH 6.0) was used for antigen retrieval which performed in a pressure cooker for 3 min. After blocked in 1% BSA for 1.5 h at room temperature, tissue sections were incubated with primary antibodies at 4°C. Then, for IF, the samples were incubated with secondary antibodies conjugated with Alexa fluor for 1 h at room temperature, counterstained with 4′,6-diamidino-2-phenylindole dihydrochloride (DAPI) to detect nuclei and visualized using fluorescence microscopy. For IHC, the secondary antibody was diluted to 1:750 for recognizing primary antibodies. The staining for IHC was visualized using the VECTASTAIN ABC peroxidase system and peroxidase substrate DAB kit.

### RNA sequence

The cutaneous wound tissues were obtained and extracted into total RNA using TRIzol® Reagent according the manufacturer’s instructions (Invitrogen). DNase I (TaKara) was used to remove genomic DNA and then the quality of RNA was examined by 2100 Bioanalyser (Agilent) and quantified using the ND-2000 (NanoDrop Technologies). The high-quality RNA sample (OD260/280=1.8~2.2, OD260/230≥2.0, RIN≥6.5, 28S:18S≥1.0, >10μg) was used for sequencing library construction.

Following TruSeqTM RNA sample preparation Kit from Illumina (San Diego, CA), RNA-seq transcriptome library was prepared using 10μg of total RNA. The raw paired end reads were trimmed and quality controlled by SeqPrep and Sickle with default parameters. Then clean reads were separately aligned to reference genome with orientation mode using TopHat software. Differential expression genes between two different samples was calculated according to the fragments per kilobase of exon per million mapped reads (FRKM) method. RSEM was used to quantify gene abundances. R statistical package software EdgeR was utilized for differential

expression analysis. In addition, functional-enrichment analysis including GO and KEGG were performed to identify which DEGs were significantly enriched in GO terms and metabolic pathways at Bonferroni-corrected P-value ≤0.05 compared with the whole-transcriptome background. GO functional enrichment and KEGG pathway analysis were carried out by Goatools and KOBAS.

### shRNA transfection

AKT3 and negative control shRNA were obtained from OBiO (Shanghai, China). The shRNA (50 nM) was transfected into THP-1-derived M2 macrophages using Attractene transfection reagent (Qiagen), according to the manufacturer’s protocol.

### RNA isolation and quantitative reverse transcription polymerase chain reaction (qRT-PCR)

Total RNA was extracted using Trizol (Invitrogen, Grand Island, NY). First-strand cDNA was synthesized using SuperScript III reverse transcriptase (Invitrogen, USA). mRNA expression was analyzed by qRT-PCR using PowerUp™ SYBR® Green Master Mix (Thermo Scientific, Waltham, MA, USA). Relative mRNA expression levels were quantified using the 2^-ΔΔCt^ method with β-actin as the internal control. The specific primers used for qRT-PCR are listed in Supplementary Table 1.

### Total protein extraction and western blotting

Cells were lysed on ice in RIPA buffer (Beyotime, Suzhou, China) containing 1% (v/v) protease inhibitor cocktail (Roche, Indianapolis, IN). For tissue proteins, tissues were homogenized and then lysed in RIPA buffer on ice for 30 min. Protein concentrations were determined using the Pierce BCA Protein Assay (Thermo Scientific, Waltham, MA, USA). Protein (20 μg) was separated on 7.5 to 12.5% gels by sodium dodecyl sulfate-polyacrylamide gel electrophoresis (SDS-PAGE) and transferred onto polyvinylidene fluoride (PVDF) membranes (Millipore, Billerica, MA, USA). After blocking with skim milk, the membranes were incubated with primary antibodies and then HRP-conjugated secondary antibodies. The specific protein bands were visualized using the ECL substrate solution (NCM, Suzhou, China) and ChemiLucent ECL Detection System (Thermo Scientific, Waltham, MA, USA).

### Cell proliferation assays

Co-cultured Cells (HSFs and JB6) (2000 per well) were plated in 96-well plates. When the control (vehicle)-treated cells reached 95% confluence, the cells received the indicated treatments. At each time point, CCK-8 reagent (20 μL) was added to each well and incubated at 37°C for 2 h. The absorption values at 450 nm were measured using a multi-well plate reader (BioTek, Winooski, VT, USA).

Cell proliferation was also examined using the Cell-Light™ EdU DNA Cell Proliferation Kit (Ribobio, Guangzhou, China). EdU (5-ethynyl-2’-deoxyuridine) was added to the cells for 2 h. EdU incorporation was analyzed by fluorescence microscopy.

### Cell migration assay

HSFs were seeded in the chambers of transwell inserts, and the bottom chambers were filled with normal control medium or conditioned medium. After 24 h, the cells remaining on the top side of the filter membrane were wiped off gently with a cotton swab. The cells that migrated to the lower surface were fixed with 10% buffered formalin, stained with 0.1% crystal violet for 10 min at room temperature, and counted using an inverted microscope (Leica, Wetzlar, Germany).

### Animal experiments

AKT3 knockout mice (AKT3^-/-^, C57BL/6 background) were generated by Model Organisms (Shanghai, China). Briefly, exons 3 to 5 were deleted from the AKT3-203 transcript using CRISPR/Cas9 technology. The sequence of the guide RNA was 3′-TGAACCAGTCAGACTGAAGA TGG-5′. The AKT3 knockout vector was injected into ES cells, and then the homologous recombination (HR) of ES cells was selected. The HR ES cells were injected into the blastocysts to generate chimera mice. The chimera mice were crossed with AKT3 wild-type mice (AKT3^+/+^, C57BL/6 background) to obtain the AKT3^-/-^ mouse strain. The cutaneous wound model was established as previously described [51]. Briefly, mice were anesthetized with ketamine/xylazine (100 mg·kg^-1^, i.p.), and the back skin was shaved and disinfected with an ethanol solution. Full-thickness excisional wounds were created using a 10-mm biopsy punch and left without cover during the wound healing process.

### Statistical analysis

Data are presented as the mean ± SEM. Statistical analysis was performed using SPSS 17.0 software (SPSS Inc., Chicago, IL, USA). Groups were compared using the two-tailed Student’s t-test or one way ANOVA. *P < 0.05, **P<0.01, ***P<0.001 was considered statistically significant. Each experiment was performed in triplicate.

## Supplementary Material

Supplementary Figures

Supplementary Table 1
