# Supplementary material for: AKT3 deficiency in M2 macrophages impairs cutaneous wound healing by disrupting tissue remodeling
Source: Aging (Albany NY). 2020 Apr 14;12(8):6928–46. doi: 10.18632/aging.103051 (PMC7202485; doi:10.18632/aging.103051)
Supplement: Supplementary Figures [file aging-12-103051-s002..pdf]

SUPPLEMENTARY FIGURES

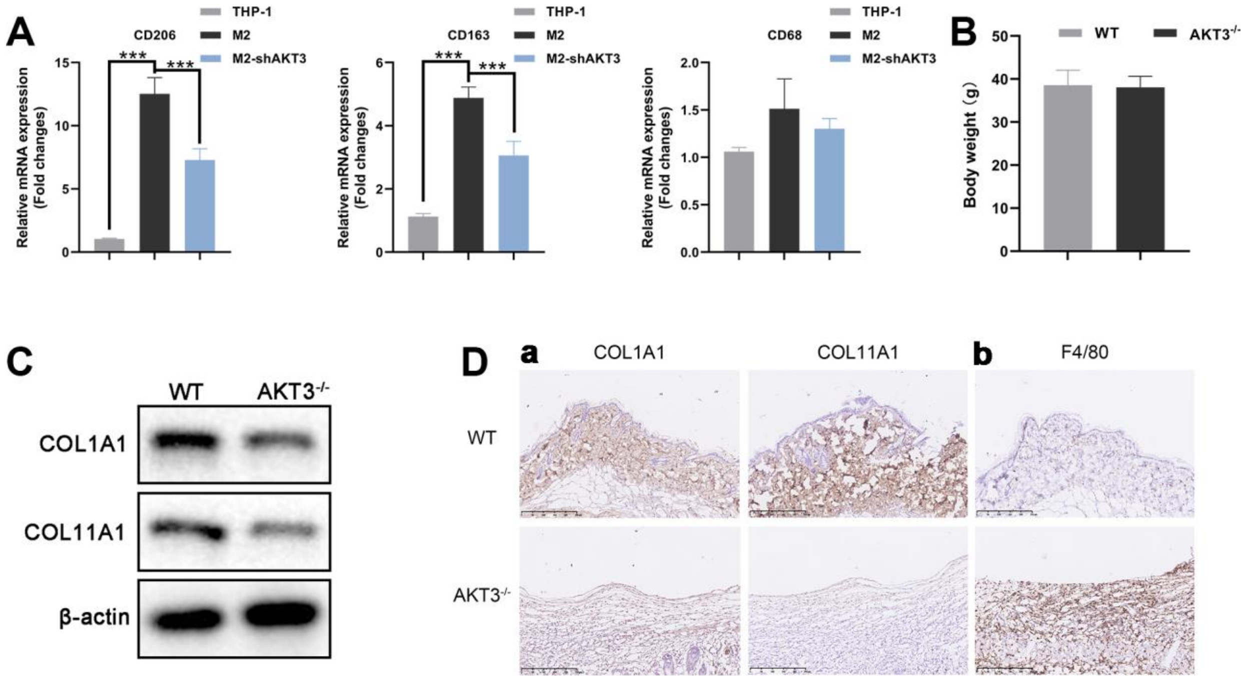

Supplementary Figure 1. (**A**) CD68, CD163, and CD206 mRNA expression levels in THP-1-derived M2 macrophages measured using qRT-PCR. (**B**) Body weights of $AKT3^{+/+}$ and $AKT3^{-/-}$ mice. (**C**) COL1A1 and COL11A1 expression measured using Western bolting in wound tissue of $AKT3^{+/+}$ and $AKT3^{-/-}$ mice. (**D**) The level of COL1A1, COL11A1 and F4/80 expression in cutaneous tissue of $AKT3^{+/+}$ and $AKT3^{-/-}$ mice detected by IHC. All the experiments were repeated at least three times.

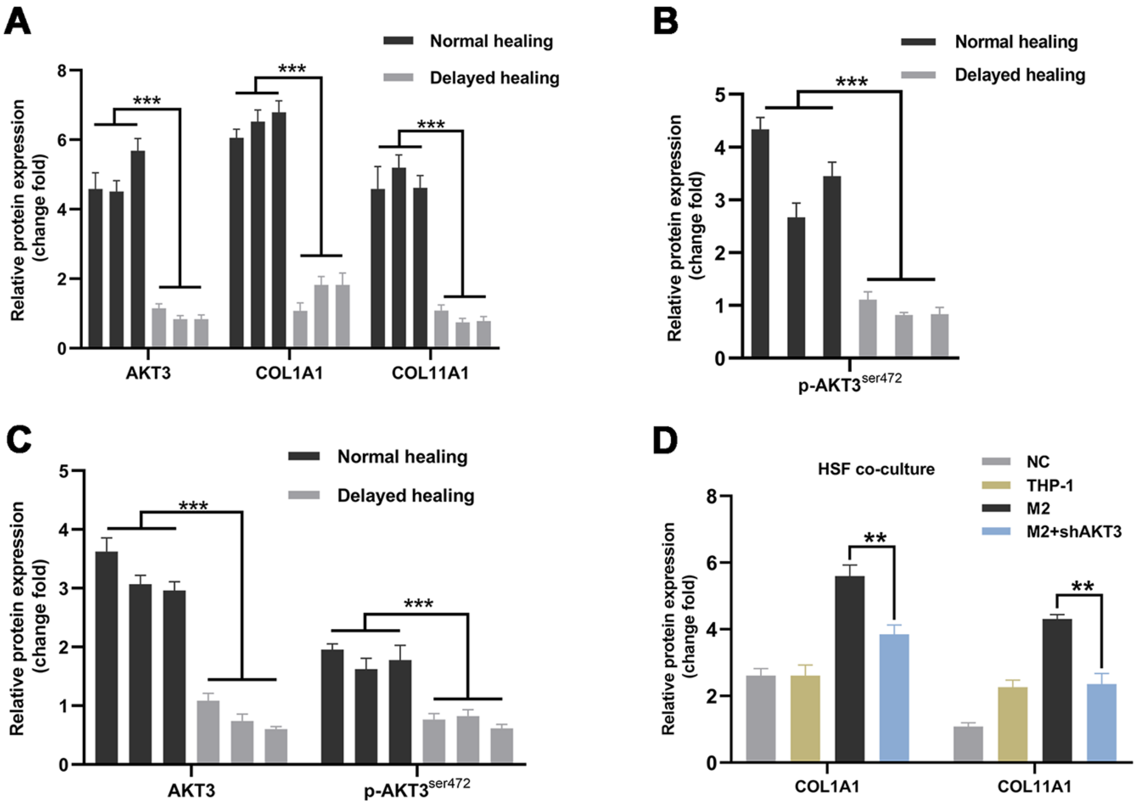

Supplementary Figure 2. (**A**) The quantified data of Figure 3E (n=3). (**B**) The quantified data of Figure 3F (n=3). (**C**) The quantified data of Figure 4E (n=3). (**D**) The quantified data of Figure 5I (n=3). All the experiments were repeated at least three times.
