# Supplementary material for: AKT3 deficiency in M2 macrophages impairs cutaneous wound healing by disrupting tissue remodeling
Source: Aging (Albany NY). 2020 Apr 14;12(8):6928–46. doi: 10.18632/aging.103051 (PMC7202485; doi:10.18632/aging.103051)
Supplement: Supplementary Table 1 [file aging-12-103051-s001..pdf]

SUPPLEMENTARY TABLE

Supplementary Table 1. Primer sequence.

Gene symbol	Forward primer	Reverse primer
AKT3	5’-TGTGGATTTACCTTATCCCCTCA-3’	5’-GTTTGGCTTTGGTCGTTCTGT-3’
TGF-β	5’-AGCTGCGCTTGCAGAGATTA-3’	5’-AGCCCTGTATTCCGTCTCCT-3’
IL-10	5’-CTTACTGACTGGCATGAGGATCA-3’	5’-GCAGCTCTAGGAGCATGTGG-3’
CD206	5’-TCCGGGTGCTGTTCTCCTA-3’	5’-CCAGTCTGTTTTTGATGGCACT-3’
CD163	5’-TTTGTCAACTTGAGTCCCTTCAC-3’	5’-TCCCGCTACACTTGTTTTCAC-3’
CD68	5’-GGAAATGCCACGGTTCATCCA-3’	5’-TGGGGTTCAGTACAGAGATGC-3’
β-actin	5’-CATTCCAAATATGAGATGCGTT-3’	5’-TACACGAAAGCAATGCTATCAC-3’
